# Adverse pregnancy outcome disclosure and women’s social networks: a qualitative multi-country study with implications for improved reporting in surveys

**DOI:** 10.1186/s12884-022-04622-1

**Published:** 2022-04-06

**Authors:** Doris Kwesiga, Leif Eriksson, Christopher Garimoi Orach, Charlotte Tawiah, Md Ali Imam, Ane B. Fisker, Yeetey Enuameh, Joy E. Lawn, Hannah Blencowe, Peter Waiswa, Hannah Bradby, Mats Malqvist

**Affiliations:** 1 Department of Women’s and Children’s Health, Uppsala University; Uppsala Global Health Research On Implementation and Sustainability (UGHRIS), Uppsala University, Uppsala, Sweden; 2 Department of Health Policy, Planning and Management, School of Public Health, Makerere University, Kampala, Uganda; 3 Department of Community Health and Behavioral Sciences, School of Public Health, Makerere University, Kampala, Uganda; 4 Kintampo Health Research Centre, Kintampo, Ghana; 5 Maternal and Child Health Division, International Centre for Diarrhoeal Disease Research, Dhaka, Bangladesh; 6 Bandim Health Project, Bissau, Guinea-Bissau; 7 Research Centre for Vitamins and Vaccines, Statens Serum Institut, Copenhagen, Denmark; 8 Open Patient Data Explorative Network (OPEN), University of Southern Denmark, Odense, Denmark; 9 Department of Epidemiology and Biostatistics, Kwame Nkrumah University of Science and Technology, Kumasi, Ghana; 10 Maternal, Adolescent, Reproductive & Child Health Centre (MARCH), London School of Hygiene & Tropical Medicine, London, UK; 11 Department of Public Health Sciences, Karolinska Institutet, Stockholm, Sweden; 12 Department of Sociology, Uppsala University, Uppsala, Sweden

**Keywords:** Social networks, Disclosure, Adverse pregnancy outcomes, Diffusion of innovation

## Abstract

**Background:**

Globally, approximately 6,700 newborn deaths and 5,400 stillbirths occur daily. The true figure is likely higher, with under reporting of adverse pregnancy outcomes (APOs) noted. Decision-making in health is influenced by various factors, including one’s social networks. We sought to understand APOs disclosure within social networks in Uganda, Ghana, Guinea-Bissau and Bangladesh and how this could improve formal reporting of APOs in surveys.

**Methods:**

A qualitative, exploratory multi-country study was conducted within four health and demographic surveillance system sites. 16 focus group discussions were held with 147 women aged 15–49 years, who had participated in a recent household survey. Thematic analysis, with both deductive and inductive elements, using three pre-defined themes of Sender, Message and Receiver was done using NVivo software.

**Results:**

Disclosure of APOs was a community concern, with news often shared with people around the bereaved for different reasons, including making sense of what happened and decision-making roles of receivers. Social networks responded with comfort, providing emotional, in-kind and financial support. Key decision makers included men, spiritual and traditional leaders. Non-disclosure was usually to avoid rumors in cases of induced abortions, or after a previous bad experience with health workers, who were frequently excluded from disclosure, except for instances where a woman sought advice on APOs.

**Conclusions:**

Communities must understand why they should report APOs and to whom. Efforts to improve APOs reporting could be guided by diffusion of innovation theory, for instance for community entry and sensitization before the survey, since it highlights how information can be disseminated through community role models. In this case, these gatekeepers we identified could promote reporting of APOs. The stage at which a person is in decision-making, what kind of adopter they are and their take on the benefits and other attributes of reporting are important. In moving beyond survey reporting to getting better routine data, the theory would be applicable too. Health workers should demonstrate a more comforting and supportive response to APOs as the social networks do, which could encourage more bereaved women to inform them and seek care.

**Supplementary Information:**

The online version contains supplementary material available at 10.1186/s12884-022-04622-1.

## Background

Globally, there is a high annual number of adverse pregnancy outcomes (miscarriages, stillbirths and neonatal deaths), many of which are preventable. An estimated 2.4 million newborn deaths (deaths in the first 28 days of life) occurred in 2019, that is 6,700 newborns dying daily [1]. Stillbirths globally were approximately 2 million, with a stillbirth rate of 13.9 stillbirths per 1,000 total births [1]. The estimates for miscarriage, mostly occurring in the first trimester, range from 11–22% among pregnancies that are detected [2].

Adverse Pregnancy Outcomes (APOs) have negative effects on parents and families, including psycho-social challenges like depression, anxiety, denial, loss of identity as a mother or father as well as uncertainty around disclosure [3–5]. With the invisibility of stillbirths in routine reporting, many of these effects are never addressed [1].

Many APOs can be avoided through appropriate health care [1]. However, evidence suggests APOs and pregnancies are under reported globally, particularly in sub-Saharan Africa and southern Asia, which have the highest burden of APOs [1, 6]. Under reporting results in public health planning based on inaccurate statistics, thus too few resources are devoted to tackling APOs. More accurate figures are key to monitoring progress towards the achievement of targets like Every Newborn Action Plan (ENAP), which seeks to reduce neonatal deaths to 12 or fewer per 1,000 live births and stillbirths to 12 or fewer per 1,000 total births globally by 2030 [7].

Decision-making and social support in health are influenced by multiple factors, including relationships with other people, according to studies on prostate cancer, maternal health and HIV/AIDS [8–10]. A network is a collection of relationships, with known description and directionality, clear connections between them, shared qualities and flow or exchange of items [11]. According to network theory, relationships around a person can both provide but also hinder opportunities and so while studying somebody’s achievements, one should not only seek to understand individual characteristics, but also the relationships around them that likely influence their choices and behavior [12]. Networks influence motivations and behavior, including health behavior, over and above the individual relationships that constitute the network.

We believe that studying social networks around the time of occurrence of APOs is important if we want to have a deeper understanding of under reporting of these events. Key people in the social networks of the pregnant woman and / or her partner may include relatives, friends and neighbors. Indeed, social networks are important influencers of decisions and behavior and APOs must be considered within the social context in which they occur [13].

Studies have shown that social networks can assist the bereaved person to cope better with the effects of APOs, including being able to talk about it [5, 14, 15]. However, not much is known about what happens within social networks when APOs occur in low- and middle-income country settings. Information, perceptions and behavioral patterns developed through social interactions are shared through social networks and may directly impact reporting of APOs. For example, a shared lack of awareness of the requirement to report perinatal deaths and a lack of knowledge of who should receive such a report have been noted as barriers to reporting [16]. In other instances, social networks play a big role in compliance or non-compliance with recommended medical action, as seen in the case of Ebola [17].

A key feature of social networks is communication and despite variations in its definition, the communication process is widely agreed to involve a sender, a receiver and a message. While early theorists viewed communication as a one-way process from the sender to the receiver, it is now more commonly expressed as actors engaged in a two-way or omni directional flow of information or dissemination of a message involved [18]. This paper considers reporting of APOs, partly guided by communication theory.

Reporting APOs can be seen as an innovation, according to the diffusion of innovation theory, which explains diffusion as the process by which an innovation (i.e. an idea, practice or object that is perceived as new by an individual or other unit of adoption) is communicated through certain channels over time, among members of a social system [19]. This theory states that when a person is presented with an innovation, they go through five main stages in the innovation-decision process: getting knowledge (about the innovation); persuasion (attitude towards it), decision (to adopt it or not), implementation (using it) and confirmation (reinforcement or reversing from use).

The novelty of an innovation implies that if it seems new to a person, then so it is, regardless of it having existed longer. Therefore, although reporting of APOs is not a new thing in surveys, it may be so to persons in communities. Newness also goes beyond having knowledge about the intervention, to having the correct attitude.

Our paper examined how and why disclosure of APOs takes place within social networks in four Health and Demographic Surveillance System (HDSS) sites in Uganda, Ghana, Guinea Bissau and Bangladesh. Communication theory guided analysis and the diffusion of innovation theory assisted with interpretation and discussion of the findings. Understanding these communication processes in social networks could provide a learning platform to improve formal reporting of APOs.

## Methods

### Study design

This was a qualitative, exploratory study, with data collected within the Every Newborn-International Network for the Demographic Evaluation of Populations and their Health (EN-INDEPTH) study. The EN-INDEPTH study aimed to inform improvements in measurement of pregnancy outcomes in population-based household surveys [20, 21].

This paper presents findings from four HDSS sites: Bandim in Guinea-Bissau, Iganga Mayuge in Uganda, Matlab in Bangladesh and Kintampo in Ghana. Although Dabat HDSS was among the EN-INDEPTH sites, it was excluded from this paper due to inadequate data on social networks and disclosure of adverse pregnancy outcomes. Focus Group Discussions (FGDs) were conducted with women aged 15–49 years who had previously been interviewed in the EN-INDEPTH survey. These FGDs explored reporting and disclosure of pregnancy and APOs, knowledge and practices after APOs, among other topics. The FGDs were preferred to indepth interviews because we aimed to understand shared social and cultural norms and meanings around disclosure of APOs, alongside associated practices, within a short time period available [22].

### Study setting

The total population was estimated at 180,000 (Bandim), 83,000 (Iganga Mayuge), 152,519 (Kintampo) and 230,185 (Matlab). Except for Bandim that has both urban and rural sites, the three other sites were predominantly rural areas. However, part of Iganga can be classified as peri-urban, and it is situated along a major highway in Uganda, which is the road to Mombasa. In Matlab, the majority of the people are Muslims, in IgangaMayuge it’s about half Muslims and half Christians, Bandim has more Christians and Kintampo is predominantly Christian.

Although the rural communities may be closer knit than urban ones, respondents were likely referring to different social networks, with each having their own, more likely to be configured around households, family, friends and neighbors, as will be shown in the results. Further details about the study sites are shown in additional file 2.

### Participant selection

Participants were purposively selected from among the pool of EN-INDEPTH survey respondents. Selected women were approached by HDSS staff in the different sites and requested to participate in the qualitative part of the study. All sites used a face-to-face approach for recruitment. Bandim supplemented this with written notices in the absence of the potential participant when the recruitment team went to their home. Iganga Mayuge and Matlab supplemented their recruitment approach with phone calls.

Selection was undertaken to ensure representation of both urban/peri-urban and rural residents where possible, as well as variation in age. While there were no drop outs or refusals among respondents in IgangaMayuge and Kintampo, in Bandim 4 out of 24 women approached did not attend the FGDs. A total of 16 FGDs were conducted, 4 in each site, as shown in Table 1.

**Table 1:** Socio-demographic details of study respondents (*n* = 147)

Characteristic	Bandim (Guinea-Bissau)	IgangaMayuge (Uganda)	Kintampo (Ghana)	Matlab (Bangladesh)
**Total number of focus group discussions**	4	4	4	4
**Total number of respondents**	29	40	50	28
**Age (years)**				
< 25 25–35 35–49 Missing	8 (27.5%) 15 (51.7%) 6 (20.6%) 0	15 (37.5%) 11 (27.5%) 14 (35%) 0	14 (28%) 25 (50%) 10 (20%) 1 (2%)	13 (46.4%) 3 (10.7%) 12 (42.8%) 0
**Study language**	Creole (Balanta in a few cases)	Lusoga	Twi	Bengali

### Data collection

The FGDs were conducted between February and August 2018 and they explored women’s experiences with survey data collection processes, as well as experiences with disclosing pregnancy and APOs, practices after APOs, amongst other topics.

An FGD guide was developed and piloted in each site with women outside the study area. The adapted guide was used to collect data in all the sites, but sites had the flexibility to make some adaptations according to the local context and to respond to the flow of each group discussion. Although the guide was developed in English, it was translated to the predominantly spoken local languages. Respondents were able to answer in the language they were most comfortable with.

The FGDs were conducted within settings that participants could easily access, including local public health facilities or other community settings, for instance under sheds and trees and at the HDSS site offices. Each respondent provided written informed consent at the start of the FGDs. Those below 16 years of age gave assent after informed consent from their parent/legal guardian to take part in the study. Length of the FGDs was between 1.5 to 2 h and for each group an audio recording was made. To supplement audio recordings, a research assistant at each site took written notes. The audio recordings were transcribed verbatim and translated into English (except in IgangaMayuge where transcription was directly into English). In addition, notes were taken by other members of the research team and these were used as supplementary material for analysis and to understand the study context.

Ethical approval for the study was granted in each country by recognized institutional review boards, as well as by the review board at London School of Hygiene and Tropical Medicine (additional file 4).

### Data analysis

Thematic analyses [22] were done in NVivo 12 software, with phase one of analysis comprising reading through the data repeatedly, understanding its meaning and creating codes. In the second phase, we read the transcripts again and then grouped sets of codes that had similar meanings and between which relationships could be established. Thereafter, groups of codes were placed under three pre-defined themes (Sender, Message and Receiver), based on communication theory [18]. Finally, under each theme, sets of codes were grouped to form sub-themes. Analysis included both inductive and deductive features. Moreover, analysis included identification of both similarities and differences in responses between sites, as well as identifying outlying views or explanations that differed from others in the group. The three investigators who led data analysis regularly had online meetings to review and refine the codes and decide how to write up the results.

Research team and reflexivity: Data collection was led by a multi-site qualitative working group, which worked with local staff and researchers to ensure quality of the interviews. All those who participated in data collection were citizens of their respective countries and native speakers of the language in which the FGD was conducted. An exception was in Bandim and Kintampo where occasionally one woman was noted as using a different dialect and her friend translated what she said for the research team.

One author moderated some of the Kintampo FGDs, while another attended all sessions in IgangaMayuge but none of the authors moderated in the other sites. Moderators were women in Kintampo and Matlab, men in Iganga Mayuge and Bandim, while note takers were men in IgangaMayuge, women in Bandim and Matlab and women and men in Kintampo. We emphasize the gender of the team members because of the possibility that it could affect the extent to which women open up due to being either more or less comfortable discussing such topics [23].

## Results

Within the pre-defined three broad themes in this study, that is Sender, Message and Receiver, we identified a number of sub-themes, as shown in Table 2 and discussed further in this section. Within these themes and sub-themes, we highlight the communication process when APOs occur. We highlight who shares the message about APOs, what the content of the message is, the process of communication, who is told and their reactions on receiving the news.

**Table 2:** Themes and sub-themes

Theme	Sub-theme
Sender	Identity of the sender
	Reasons for disclosure of the adverse pregnancy outcome
Message	Content of the message
	How the message is delivered and why
Receiver	Identity of the receiver
	Why specific people are informed about an adverse pregnancy outcome
	Reaction of network members on receipt of the news about an adverse pregnancy outcome

### Sender

#### Identity of the sender

We found that when an adverse pregnancy outcome occurred, the news was often shared by the woman who suffered the event, her partner, or their family members, including their own parents, aunts, uncles and in-laws. In a number of cases, friends disclosed it to other people around the woman.

### Reasons for disclosure of the APOs

Community expectations and practices played a role in telling other people about the occurrence of APOs. Across the different countries, it was common for people to share the outcome of a birth or to ask about it, having known that the woman had been pregnant. In many cases, it was also common for the news of APOs to be shared, in the same way that news of a living and healthy baby was communicated.

Disclosure of APOs was not necessarily undertaken by those who suffered the loss themselves, since they, and particularly the woman who had been pregnant, were said to be too distraught and so people would speak on their behalf. Therefore, disclosure was sometimes made by people other than the parents who had suffered APOs, due to concern for the well-being of the bereaved woman. For instance, respondents revealed that they informed other people about the news so that potential visitors would be warned against disturbing the woman during her time of loss, by asking her questions about the outcome of her birth when the baby had died. However, in other cases, friends or relatives had not been requested to relay news of APOs, but simply did so as part of general discussion or gossip.

What I will say is, it is not as if you broadcast it to everyone, but then your family members will tell your close friend and the man that you have lost the baby. So that later in the future, no one will come and question you as to what happened to the baby (Kintampo, Ghana).

Making sense of what happened and trying to understand why APOs occurred was influential in disclosure of APOs to others within one’s social networks. For instance, in a few cases people informed health workers about APOs because they believed in their advice or because they wanted the health workers to provide insights into the cause of the baby’s death and how future adverse pregnancy outcomes could be avoided. Furthermore, a few respondents mentioned that sometimes people disclosed APOs when they occurred repeatedly and the woman was therefore desperate.

If they [APOs] happen more than once, then one might be forced to share with village or clan members in order to get a solution (IgangaMayuge, Uganda).

Seeking meaning was further demonstrated by the family looking for solutions beyond the health facilities. In one case, a respondent explained that if one side of the family was not satisfied with the health worker’s explanations, this resulted in counter-accusations about who was responsible for the death and therefore they sought alternative explanations through other cultural rituals.

Those babies that die in the stomach are brought to be buried, those that are born and then die after, other races/tribes say that they are going to find out what it was that killed the baby. Like in the Manjaco tribe, they can say we are going out to … see who killed the baby, to know if it was the guy’s family or the woman’s family who managed to kill him…. (Bandim, Guinea-Bissau).

Personal responsibility for taking the initiative to inform the health workers also prompted disclosure. A few respondents said that health workers had to be informed that the woman or baby would not be returning for care, while others informed health workers who were their friends or relatives.

Yes, you will have to inform them [the health workers]. Maybe you had a stillbirth and you have to come and inform them [the health workers] so that they will know that you are no longer among the pregnant women (Kintampo, Ghana).

Nevertheless, there were also instances where people did not reveal adverse pregnancy outcomes within their social networks. The need to avoid blame and rumors was critical here, especially with the stigma often attached to induced abortions. These were usually hidden, with people fearing accusations of murder or being a bad person if they confessed to an induced abortion. There was, however, a respondent who said it was easy for her to disclose having had an induced abortion, but this was an outlier and her response was not probed. With regard to informing health workers, which was among the topics in the FGDs, a respondent pointed out that they lived with these health workers in the same communities and so they would find out about the loss even without the mother herself informing them. More so, a few people mentioned that in cases where APOs occurred outside the health facilities, they did not report this back to the health workers.

Part of the reason for non-disclosure was the disillusionment with health services, where somebody previously had a negative experience in their interaction with health workers. They therefore did not see the purpose of updating the health workers about the APOs, considering their previous reception when they or their children had visited the health facility.

I don’t think it is important because when I delivered at home and sent my baby to the hospital to be weighed, they [health workers] refused to attend to me so when something like this [APOs] happens in the home I would not even report it to them (Kintampo, Ghana).

### MESSAGE

In this theme, we focus on the message about the adverse pregnancy outcome, including its content, delivery and the receiver’s verbal response towards the message. We describe the content from both the side of the sender and the receiver, according to what was said. Additionally, we discuss the delivery of the message, including whether disclosure was done directly or indirectly and its timing.

### Content of the message

There were both direct and indirect ways of describing the occurrence of APOs. Directly, the loss was described unambiguously as the occurrence of a death. Indirectly, sometimes the actual names used to describe APOs in the different sites were metaphorical, not directly speaking about the loss of a baby. For instance in Balanta villages in Guinea-Bissau, as well as in the Creole language, “Auor” (give birth to but not to have) is one of the ways in which a baby born dead is described, while in Kintampo, “w’apon ayinsen” or “ayinsen no aseɛ” (the pregnancy is finished or terminated or spoilt), describes a miscarriage and “w’awo atwene” (the woman has given birth and thrown it away) refers to a stillbirth.

We identified two broad categories of the verbal message after APOs: a message of comfort and a message of blame. Primarily, people in the social network responded to news of APOs with words of comfort, sympathy and encouragement to the bereaved woman or family, as well as advice on what to do. Across all the sites, messages based on religion were directed at the need for the bereaved to focus on a higher power as the provider of life. In a few cases, the response was packaged as a message of accepting inevitability, about which nothing could be done, but such fatalism was nonetheless accompanied with words of encouragement.

… It’s not in our hand. It [APOs] is imposed by fate or Allah’s wish, that Allah didn’t want it. If Allah wanted, it would have survived. People would say this (Matlab, Bangladesh).

On the other hand, there were a few cases where the message was one of blame and finger pointing, with negative things said once an adverse pregnancy outcome occurred. These included criticism and blame of the mother for having done the “wrong” things before or during her pregnancy, either health wise or culturally. There was also criticism of both parents for not looking after the newborn correctly. In a few cases, APOs were attributed to somebody having bad luck. A few respondents mentioned the gossip that took place after repeated occurrence of APOs.

This generation, girls go for family planning and we hear that the eggs get weakened by the drugs. The fetus may turn to be a miscarriage. So, sometimes, the woman is blamed (IgangaMayuge, Uganda).

### How the message is delivered and why

Direct disclosure was often within familial, friendship and hierarchical relationships, where people were directly informed of the loss by the woman herself or her partner. Direct disclosure to non-clan or non-family members was mentioned by a few respondents in IgangaMayuge as being done to seek advice when the APOs were repetitive.

Indirect disclosure involved people learning about the loss from a family member, rather than the woman that had experienced the event or her partner. These people disclosed to were often the neighbors and sometimes the close friends or even the father of the baby as mentioned in one FGD. Furthermore, in one case, a respondent said that by virtue of the fact that people had seen you pregnant, they eventually found out about APOs since they could see you had returned without a baby. In this way, the news was not deliberately shared but inevitably got to be known. For both direct and indirect disclosure, the message was often delivered verbally and in one site, telephones were mentioned as a means of communication.

It is neither kept hidden nor spread to everyone. For example, my child is lost (miscarriage), those who know, people who live around, asked, did you have a baby? Got lost or where was it? I was in the hospital, why was I there? These are the problems. It’s not like all people have to know or you have to inform all. It is not like you have to inform all by calling. It has been lost (miscarried), that’s all (Matlab, Bangladesh).

However, in Kintampo, among the cultural practices described was shaving the heads of the people who suffered APOs, as an indication of the loss. This partly showed that one was bereaved and so people did not need to ask them about the baby. Nevertheless, it was also mentioned that this practice was rarely done now.

Lastly, disclosure of APOs within the social networks happened at different times. Sharing the news was not necessarily always done as soon as it occurred. For instance, sometimes while the woman was still in hospital, the news had already been relayed to other friends and neighbors. With specific reference to women who had repeated APOs, it was mentioned in a few cases that this news would be shared only after repeated occurrences.

### The receiver

The sub-themes here describe the identity of the receiver, their role or position in society that places them in line for receiving the news of APOs and what happens when they do, beyond the content of the message already discussed.

### Identity of the receiver

In this study, we found that various people in one’s social network received the news of the death, including biological family, comprising parents, siblings and other close relatives like aunts and uncles and marital family, as well as the husband/father of the baby, together with the in-laws. Other social network members comprised friends, neighbors, other community members including clan members and village mates, religious leaders, clan heads and occasionally health workers. The elders were part of the social networks in various sites, while specifically for Kintampo, the chiefs (or any elderly person who represented the chief) and the landlord were spoken about.

### Why specific people are informed about an adverse pregnancy outcome

Proximity and convenience played a predictable role in determining who was told about APOs, with both physical and emotional proximity to the bereaved having an influence. In all countries, it was revealed that people who had APOs most commonly disclosed mainly to those around them including family, especially friends, those with whom they shared a house and sometimes close neighbors. In some instances, these people were already present at the time APOs occurred and so were aware of it, or were informed later. Inevitably also, these were people with whom relationships had been built, thus they confided in them. In a few cases, respondents said that news of APOs was only shared with family members, especially the earlier gestation ones like miscarriages.

Our analysis shows that the roles played by certain people in a community are the reported reason why they received information about APOs. For instance, some receivers were often regarded as decision-makers. These were particularly the men (including the woman’s husband or partner), the elders and cultural leaders. The husband or partner was mentioned when the respondent in the FGD was explaining whom the women disclosed to, rather than whom the couple (of both husband and wife) disclosed to. Secondly, among the major roles this group of decision-makers played was planning how to deal with the situation and handle the burial. Men were often in charge of this process, however small or informal the burial. If the husband or partner was not around, in a few cases it was reported that the woman’s father was consulted, as well as cultural leaders.

If you have an important man at home, or you can go out and look for other important men to tell who comes to “cover me” [to bury the dead] (Bandim, Guinea-Bissau).

Maybe the father of the child [can perform the burial arrangements], but in his absence the father of the woman can do that (Kintampo, Ghana).

Furthermore, providing spiritual leadership in key religious practices like leading funeral prayers was among the criteria for being informed about APOs. Religious leaders, including pastors, sheiks and imams were noted as conducting prayers and also comforting the bereaved. Indeed, prayers for the deceased were frequently mentioned. Additionally, in two sites a few people mentioned traditional rites in addition to funeral prayers.

The religious leaders do conduct prayers [after APOs]. This depends on the religious affiliation of the family. But religious leaders do console the bereaved unlike traditional leaders who go to shrines and carry out their rituals. The religious leaders console the bereaved family saying that it is God who gives and he takes away our loved ones (IgangaMayuge, Uganda).

Gender roles influenced who received information about the death. For instance, specific burial roles were allocated depending on the sex of the baby. Women in Matlab were charged with bathing and preparing the female babies for burial, while the men dealt with the male babies. In Kintampo, elderly women were reported to have the responsibility of preparing the body for burial. In all sites, men were the ones mentioned as leading the religious ceremonies. The burial-related gender roles in the other two sites were not mentioned. However, women were often reportedly engaged in providing emotional and social support to the family after APOs across the four sites.

Community hierarchies were also evident; for instance, there were certain people for whom it was essential that they be told, including the traditional leaders.

We inform the chief or any elderly person who represents the chief [when APOs occur] (Kintampo, Ghana).

### Reaction of network members on receipt of the news about APOs

Support and comfort to the bereaved woman and man by members in the social network were consistently shown across all the sites. Support could be moral or emotional, for example consoling and comforting those who suffered APOs, encouraging them and offering sympathy. It was frequently reported that the woman was surrounded with people to ensure she was not lonely and to help her cope with the loss. While such support was often received from friends, family and community members as mentioned in all study sites, in IgangaMayuge a few respondents mentioned health workers as also offering sympathy to the woman. In addition to company, sometimes the woman found a place of physical solace, for instance at her parents’ home.

We just make sure she [mother who had APOs] is never alone. We console her, chat with her and make her lively so that she will not be thinking about her loss (Kintampo, Ghana).

Other support was in kind, for instance some people came to cook meals for the family that had APOs, or bought them food and drinks, while others helped the woman with physical activities like bathing. It was pointed out in different sites that sometimes people took the initiative to come and ask what help they could give once they heard about the loss. In one FGD in IgangaMayuge, a few people reported that organisations (they did not clarify which type) supported people with condolences and counselling after the occurrence of APOs. Provision of financial support was important in cases where burial was done and so the networks played a role here through fundraising or making financial contributions for the family. Additionally, they helped with provision of medical attention, including taking the woman to the health facility or in the case of health workers, providing the actual medical care.

I was 4 months pregnant in Bissau, when I went to the neighbourhood of Missira. When I went to my uncle’s house, I went to the bathroom and I washed my body and my womb started to come out. No-one else was up there, only my uncle’s son, a small boy that I prepared for school. I called my uncle and told him. When he arrived, he took me to the health centre where the health workers finished up doing everything (Bandim, Guinea-Bissau).

Planning and conducting the burial, including making arrangements was done by the social network members. Additionally, they were said to be helpful in conducting the required cultural practices like tying a thread and needle to the woman’s breast to prevent the baby from returning to cause harm.

## Discussion

Overall, there was a lot of similarity in disclosure to social networks across the sites, despite their contextual differences. For instance, the message of comfort and the offer of social and other support on hearing about APOs, as well as the stigma towards induced abortion and the central role of men as decision makers. A few outstanding differences were the seemingly higher acceptability of reporting APOs to health workers in IgangaMayuge and disclosure of APOs to chiefs or their representatives in Kintampo. In Bandim, there was mention of blame between families where the cause was unclear, while in Matlab we saw intricate division of gender roles based on religion and mention of disclosure of APOs by telephone. Despite indicating some differences in the results section, overall we analyzed the data from the different sites as one data set.

It is known that APOs are under reported, in addition to most formal reporting being done around stillbirths and neonatal deaths (and not miscarriages), as shown in an earlier paper on barriers and enablers to reporting in the same countries, which identified a dose–response to reporting, with deaths of later gestation more likely to be reported [24]. However, reporting of APOs is being done informally within these networks and the current paper shows the why, whom and how of this. In the same previous study on barriers and enablers, we recommended that to improve formal reporting in surveys, the focus should be on the interviewer and their skills, as well as improved tools and local adaptation of these [24]. Data used in that study included both the women and interviewers, while in the current study with women only, we further recommend focus on the respondents, their social networks, and the reporting itself since people are not silos, but actually exist within social networks that influence their behavior and beliefs. How then can the causes and effects of APOs, together with why they should be formally reported, be communicated via networks?

We use the diffusion of innovation theory [19] to guide us on how information flows in a community and how people adopt innovations and we link this to formal reporting of APOs (See additional file 1 for schematic of the theory). It places people into different categories depending on their rate of adoption. This includes innovators (active information seekers and first users), followed by early adopters, early majority, late majority and laggards. While the innovator category typically learns about the innovation from the change agent or from channels like mass media, most of the other categories learn from the subjective evaluation of their peers’ experiences, who then become role models.

Furthermore, it explains that innovations have key attributes, which are its relative advantage (does the person see it as beneficial); compatibility (with people’s norms and values); complexity (how easy or difficult it is to do/understand); trialability (can one try it out first) and observability (are the outcomes visible). The higher the advantage, compatibility, trialability, observability and ease of use, the more likely the uptake.

Improving survey reporting may require that firstly, communities understand why they must report, to whom and where. This includes recognizing why APOs are important, their causes, effects and why they should be formally reported, as well as understanding broader maternal, newborn and child health (MNCH) information. By looking at formal reporting of APOs as an innovation, the diffusion of innovation theory would help in community entry and engagement for example before the start of a survey, to sensitise people about the survey purpose and benefits of truthful reporting. This is because the theory highlights the role of opinion leaders, who are those able to informally influence behavior and beliefs of people within their social system. In our study, we identified key gatekeepers who hold power, some of whom may not traditionally be included in MNCH programs, especially chiefs, religious leaders, elders and men. These can be approached with the aim of supporting the diffusion of information about reporting APOs within their communities during the entry stage. Although reporting of APOs in the demographic and health surveys is part of the women’s questionnaire, we have seen the decision-making role of men that can be positively utilized to change the culture of non-reporting by women. Gatekeepers can be instrumental in mobilizing the population to take up recommended actions like formal reporting of APOs, since they are respected and trusted. Additionally, if we went beyond survey reporting to getting better routine data, the theory would be applicable too.

Closely linked to this is the concept of homophily, which highlights that people tend to gather together with those with whom they share certain characteristics or similarities including gender, ethnicity, beliefs, family networks, job networks and others [8, 19]. Homophily has been found to influence health behavior of individuals, for instance smoking among older adults and decision-making around prostate cancer treatment [8, 25]. It has also been suggested that information may be better received when delivered by somebody of the same ethnicity [19], hence the benefit of the aforementioned gatekeepers.

Furthermore, in devising interventions to cause change and increase acceptability of reporting, which could be a longer term approach, one can first consider at what stage a person is in decision-making, what kind of adopter they are and their take on the benefits, ease and other attributes of reporting. Additionally, we need to understand the needs of the people we are targeting, considering their adopter category. Some may benefit from learning from the media, change agents, or peers and role models. Use of mass media has been noted as important in spreading knowledge and could be useful but the actual persuasion and learning is within the interpersonal networks [19]. Similarly, community sensitization and awareness drives could be more supplementary, active ways of reaching out to implement change and overcome barriers.

The comforting response and social support from the community on occurrence of an adverse pregnancy outcome is partly why people disclose within social networks. Therefore, health workers can learn from the social network’s response and offer humane and respectful care after APOs, including offering comfort and psycho-social support after APOs. This compassionate response could then encourage more people to actively report APOs, including those occurring outside the health facility. This may require inclusion of bereavement counselling and training on disclosure of deaths within health workers school curricula.

### Methodological considerations

Trustworthiness of research findings: We highlight the transferability and credibility of our findings, as well as study strengths and limitations. For transferability, our study was conducted in different countries in Africa and Asia, including both rural and urban settings. We had respondents of diverse ages, religion and ethnicities. Findings were similar across all four sites, so this is suggestive of generalizability of our work.

We partially show credibility by the FGDs being of sufficient length for the research teams to get adequate data. We also spent a number of months reviewing the data, thus had adequate time to get deeply familiar with it. Furthermore, we conducted investigator triangulation, with three researchers making the major decisions on the meaning of the data and development of themes.

Strengths and limitations: Data for this paper were drawn from four countries, thus a multi-country perspective from different settings. Having a large data set (one unit of analysis) with a general agreement is an advantage and increases the possibility for transferability of results.

Moreover, the FGDs gave us insights into the community level practices, which can be a resource during health planning. Furthermore, we used the same methods and tools across the different sites, both of which encouraged comparability of results.

This study was however limited by the fact that we did not set out to do a full social network analysis using the traditional methods. However, in the FGDs we collected information on social networks that helped us understand what happens within communities when APOs occur. Additionally, we did not do a rural–urban comparison of informal reporting, or assess reporting by type of APOs. Finally, we only spoke to women about these social networks. This study could have been stronger with also having FGDs with the other groups mentioned as important decision-makers. Possible next steps could include discussions with these groups to get their views on reporting of APOs and how to improve reporting in surveys.

## Conclusion

Informal reporting among social networks does not automatically translate into increased formal reporting in surveys. Deliberate efforts to improve reporting in surveys must include the community and health workers as key partners. We recommend an approach guided by the diffusion of innovation theory to bring gradual changes in the communities with regard to reporting APOs, starting with people appreciating why the need to report, supported by collaboration with opinion leaders and exploitation of interpersonal networks. Such an approach would need to be context specific and multi-pronged, because even neighboring villages have different structures and norms and people could be at different stages of adoption.

We believe community engagement will increase visibility of APOs and reduce the associated stigma. Without this type of work, governments shall continue to plan for maternal, newborn and child health services based on inaccurate numbers, to the detriment of the citizens.

## Authors' information

Not applicable.

## Supplementary Information

**Additional file 1.**

## Data Availability

The datasets generated during the current study are deposited online at https://doi.org/10.17037/DATA.00001556 with data access subject to approval by collaborating parties.

## Abbreviations

APOs: Adverse Pregnancy Outcomes
DHS: Demographic and Health Survey
ENAP: Every Newborn Action Plan
EN-INDEPTH: Every Newborn-International Network for the Demographic Evaluation of Populations and their Health
FGDs: Focus Group Discussions
HDSS: Health and Demographic Surveillance System
MNCH: Maternal, Newborn and Child Health
UN-IGME: United Nations Inter-Agency Group for Child Mortality Estimation
UNICEF: United Nations Children’s Fund
WHO: World Health Organisation
