# Supplementary material for: Adverse pregnancy outcome disclosure and women’s social networks: a qualitative multi-country study with implications for improved reporting in surveys
Source: BMC Pregnancy Childbirth. 2022 Apr 6;22:292. doi: 10.1186/s12884-022-04622-1 (PMC8988398; doi:10.1186/s12884-022-04622-1)
Supplement: Supplementary file 1 — Additional file 1. [file 12884_2022_4622_MOESM1_ESM.docx]

**Additional File 1: Stages in the innovation-decision process**


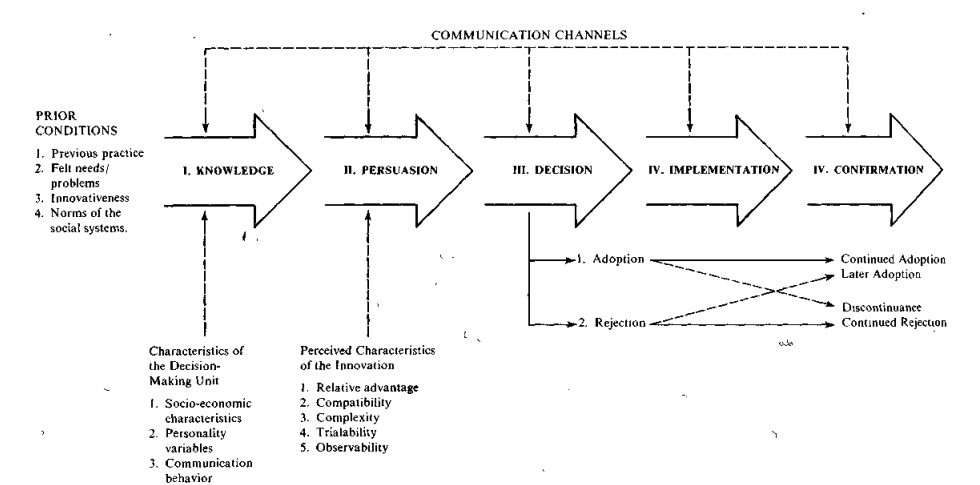


*Adapted from Diffusion of Innovations (1)*

## Additional file 2: Background details of the Health and Demographic Surveillance System sites in the EN-INDEPTH study

|  | **Bandim (Guinea-Bissau)** | **IgangaMayuge (Uganda)** | **Kintampo (Ghana)** | **Matlab (Bangladesh)** |
| --- | --- | --- | --- | --- |
| **Country** | Guinea-Bissau | Uganda | Ghana | Bangladesh |
| **Residence** | Rural and urban | Rural | Rural | Rural |
| **Population** | 180,000 | 83,000 | 152,519 | 230,185 |
| **Livebirths (per year)** | 5,790 | 2264 | 4710 | 4863 |
| **Stillbirths (per year)** | 297 | 45 | 86 | 92 |
| **Stillbirth rate, per 1000 total births (95%CI)** | 49 (43.6-54.9) | 19 (14.7 – 24.2) | 18 (14.4 – 22.3) | 19 (15.3 – 23.2) |
| **Neonatal deaths (per year)** | 204 | 118 | 95 | 104 |
| **Neonatal mortality rate, per 1000 total births (95%CI)** | 35 (30.5 – 40.1) | 52 (43.3 – 62.1) | 20 (16.2 – 24.4) | 21 (17.1 – 25.4) |

**Additional file 3: Discussion guide for the women’s FGDs**

**FOCUS GROUP DISCUSSION GUIDE FOR WOMEN: ENAP_INDEPTH SURVEY**

Interviewer ID **___ ___ ___ ___**

Note taker ID **___ ___ ___ ___**

Interview date: **__ __ / __ __ / ___ ___**

**(***DD/MM/YY***)**

Number of women in the FGD**___ ___ ___ ___**

Location of FGD**___ ___ ___ ___**

**Introduction:**

- Introduce yourself and the note taker
  - Ensure that the informants are comfortable, have time, and are able to participate in the FGD.
- Provide information about the study. Review the purpose of the FGD
  - Explain to the informants what to expect of the FGD
- Explain to the informants that:
- You especially want to learn about their experiences and thoughts on this topic
- The themes that emerge in this group will be put together with those from other groups to help get a wide understanding of what people think about this topic.

*Consent, Anonymity and Recording*

Read the consent form and obtain the participants ‘consent to proceed with the FGD. DO NOT proceed without informed consent. Ask for permission to record the interview. Assure the participants that despite being recorded, you would like to promise them that the discussion will be anonymous. The recordings will be kept safely until they are transcribed word for word, then they will be destroyed. The transcribed notes of the FGD will contain no information that would allow individual subjects to be linked to specific statements. Participants should try to answer as truthfully as possible. Nobody should discuss the comments of other group members once the FGD ends. If there are any questions or discussions that one does not wish to answer or participate in, they do not have to do so. However, they should please try to answer and be as involved as possible.

*Set the ground rules*

- The most important rule is that only one person speaks at a time. There may be a temptation to jump in when someone is talking but please wait until they have finished.
- There are no right or wrong answers
- You do not have to speak in any particular order
- When you have something to say, please do so. There are many of you in the group and it is important that I obtain the views of each of you
- You do not have to agree with the views of other people in the group
- Does anyone have any questions? (answers).
- OK, let’s begin

Start the audio recorder.

Time interview started: : **:**

- Draw a diagram of the seating arrangement.
  - Give each participant a code; Identify the seating of the participants in relation to the facilitator and recorder using the code

**Introduction**

***Start by explaining the difference between the HDSS and the ENAP-INDEPTH survey: The HDSS refers to (explain as per location, name, whom they interview, and frequency of the surveys…..***

***The ENAP-INDEPTH survey was done in the past few months by other people (in some sites by the HDSS), who came and only interviewed women. They were using a tool like the one used by the DHS (the big survey usually done by Government once in a few years)***

**Part A: Experiences with the HDSS data collection process**

Now we are going to briefly discuss the questions which the HDSS asks and the information they collect when their interviewers come to your communities. We would like to find out your thoughts.

1. Sometimes in this community, interviewers from the HDSS come to ask you questions about you and your family. These questions include asking about whether anyone is pregnant and the health of your children. Are you comfortable with questions they ask about your pregnancies and your children?

- **Probe:** What are some of the things you like about the questions they ask? What are some of the things that you are not comfortable with concerning the questions they ask?

**Part B: Experiences with the survey data collection process (ENAP-INDEPTH survey)**

Next, we would like to talk to you about the survey that just ended (ENAP – INDEPTH survey, not HDSS).

1. Some interviews have just been done, where they only talked to women, unlike the usual interviews by the HDSS. What do you think about the just ended interviews that only interviewed women? What did other women in this community think about these interviews?

- **Probe:** Time spent; convenience; questions asked; how male partners related to females being spoken to alone

1. **Additional question:** During the interviews/survey that just ended (that only involved women), they were asked questions in 2 different ways:
2. Some were asked about every time that they had ever been pregnant in their life (even if the pregnancy ended early or the baby was not born alive).
3. Other women were only asked about all their babies that were born alive and then asked separately about any stillbirths or miscarriages in the last 5 years.

In this community, do you think that it is the same from a woman’s point of view, to talk to an interviewer about:

1. Every time that a woman had ever been pregnant in her life (even if the pregnancy ended early or the baby was not born alive), compared to
2. Talking about all her babies that were born alive and then asking separately about any stillbirths or miscarriages in the last 5 years.

What is the benefit of each method? What is the disadvantage of each method? Why?

**Part C: Reporting and disclosure of pregnancy**

In this section, we are going to discuss how women in this community share information about their pregnancy, and how other people around them respond. We would like to find out your thoughts on some questions we have.

1. In this community, when a woman thinks she is pregnant who does she tell first?

- **Probe:** Who else does she tell?

1. When does she tell other people? Why does she wait/ not wait to tell other people in the community? What are the challenges that stop people from talking about pregnancy in this community?

- **Probe:** Social, religious and cultural issues; talking to strangers; what do they answer if directly asked about pregnancy

1. During the interviews/survey that just ended (ENAP-INDEPTH survey that only involved women), did you face any challenges talking to the interviewers about your pregnancies? What made it difficult to talk about this? Did any things make it easier? If yes, please explain which ones.

- **Probe:** Social, religious and cultural barriers; is it easier to talk to a man or woman; is it easier to tell a stranger than people you know?
- **Probe:** What about if the baby is stillborn? Why is it important / why is it not important?

**Part D: Reporting / disclosure of** **adverse pregnancy outcomes (neonatal deaths, stillbirths, miscarriages and / or abortions)**

Next, we are going to discuss a topic that is sad. Sometimes, a woman may be pregnant but unfortunately, the baby dies. The baby may die in the first months of the pregnancy, later on after seven months, or while she is giving birth. The baby can also be born alive, but dies before one month has passed. This may even have happened for some of you. We now want to talk about these deaths. It is a sad topic, but it will later help us to know why the babies die and what we can do to save their lives.

1. In this community, when babies die, do you have a name for this type of death? (probe for neonatal deaths, stillbirths, miscarriages and / or abortions)

Do people talk about it or do they keep quiet? Is it different if a baby is stillborn compared to if it is born alive and then dies? (if so how? /why?)

- **Probe:** Do the mothers talk about it? Whom do they talk to; when? Does the family talk about it to non-family members? (Probe for differences in reporting neonatal deaths, stillbirths, and miscarriages and / or abortions)
- **Probe:** Why do people talk about the deaths or not; what do you think makes it hard to report these deaths among babies? (In the usual HDSS interviews; in the just ended survey; in the community)? What makes it easier for women to talk about it?
- **Probe:** Socio-cultural; legal; religious; economic barriers / enablers; would it be easier or harder to talk to a man or woman; somebody you know or a stranger?
- **Probe:** Do you think it is important to report these deaths to health authorities? Why or why not?

**Part E: Gestational age and Birth weight**

In some cases when a woman is pregnant, babies are born too early or too small. We would like to learn from you a few things about this topic.

1. In this community do people think that it is important to count gestational age (from the first day of the woman's last menstrual cycle to the current date/ date when last pregnancy ended?)

- **Probe:** Why is it important? /Why is it not important? If it is important, is it easy for women to count this in this community? Why/ Why not?

1. In this community, do people think that weighing babies when they are born is important?

- **Probe:** Why is it important/ why is it not important? If it is important, how does a mother find out her baby’s birthweight in this community? Are there any problems with getting a baby weighed?
- **Probe:** What about if the baby is stillborn? Why is it important / why is it not important?

**Part E: Knowledge and practices around adverse pregnancy outcomes (neonatal deaths, stillbirths, miscarriages and/ or abortions)**

1. When a baby dies soon after birth in this community, what happens?

Is it different if a baby dies a few weeks after birth? (if so how?/why?)

What happens when a baby is stillborn? Is it different then? (if so how?/why?)

What about if there is an abortion? Is it different then? (if so how?/why?)

(First ask about the neonatal deaths, then stillbirths, miscarriages and/or abortions). For each outcome, probe as below

- **Probe:** Mourning and burial practices; community reactions; spiritual issues; practices of the family members, practices at the health facility if the baby died there; reaction of the family especially partner and in-laws; counselling for families; other support measures for victims
- **Probe:** Which people make the decision on how to deal with the death? Role of women; cultural leaders; traditional healers; traditional birth attendants; religious leaders; any other key stakeholders

1. In such situations, what happens to the women who lose their pregnancy/baby? Is it different if a baby is stillborn compared to if it is born alive and then dies? (if so how?/why?)

- **Probe:** Response of family; community; blame of the mother and stigma; empathy or support; what about those to whom it happens more than once?
- **Probe:** How do the mothers cope during this time? Role of community, healthcare providers, other organizations

**Part F: Recommendations**

Finally, we would like to conclude our discussion by asking for your advice.

1. In your opinion, what do you think can be done so that during surveys (like ENAP-INDEPTH survey and HDSS rounds), the interviewers can get better information on pregnancies, births and babies who unfortunately die?

- **Probe:** Who do you think are the best people to collect this kind of information? Why?

**Time FGD ended: ___ ___ ___ ___ ___**

*Thank the participant for their time. Remind them that the information will be kept confidential.*

**Additional File 4: Ethical approval of local institutional review boards**

| **Site** | **Institutional Review Boards** | **Date** | **Number/Ref** |
| --- | --- | --- | --- |
| Bandim (Guinea-Bissau) | Comité Nacional de Ética na Saúde | 12 June 2017 | 072/CNES/INASA/2017 |
| IgangaMayuge (Uganda) | Mildmay Uganda Research Ethics Committee  Uganda National Council of Science and Technology | 26 June 2017  11 October 2017 | REC REF 0305-2017  SS 4244 |
| Kintampo (Ghana) | Kintampo Health Research Centre, Ghana Health service Scientific Review Committee (SRC)  Ghana Health Services Ethics Review Committee  Kintampo Health Research Centre Institutional Ethics Committee | 14 June 2017  26 July 2017  9 August 2017 | SRC/130617  GHS-ERC:19/06/14  KHRCIEC/2017-14 |
| Matlab (Bangladesh) | Icddr,b Ethical Review Committee | 19 July 2017 | PR-17049 |
| London School of Hygiene & Tropical Medicine (United Kingdom) | London School of Hygiene & Tropical Medicine | 24 May 2017 | 12218 |

**References**

1. Rogers E. Diffusion of Innovations. 5th Edition. Simon and Schuster; 2003.
